# Implementing brief and low-intensity psychological interventions for children and young people with internalizing disorders: a rapid realist review

**DOI:** 10.1093/bmb/ldad001

**Published:** 2023-01-28

**Authors:** Anna Roach, Sophie Cullinan, Roz Shafran, Isobel Heyman, Sophie Bennett

**Affiliations:** UCL Great Ormond Street Institute of Child Health, 30 Guilford Street, London WC1N 1EH, UK; Institute of Education, UCL's Faculty of Education and Society, University College London, 20 Bedford Way, London WC1H 0AL, UK; UCL Great Ormond Street Institute of Child Health, 30 Guilford Street, London WC1N 1EH, UK; UCL Great Ormond Street Institute of Child Health, 30 Guilford Street, London WC1N 1EH, UK; UCL Great Ormond Street Institute of Child Health, 30 Guilford Street, London WC1N 1EH, UK

**Keywords:** implementation, brief and low-intensity interventions, children and young people

## Abstract

**Introduction:**

Many children fail to receive the mental health treatments they need, despite strong evidence demonstrating efficacy of brief and low-intensity psychological interventions. This review identifies the barriers and facilitators to their implementation.

**Sources of Data:**

PsycInfo, EMBASE and Medline were searched and a systematic approach to data extraction using Normalization Process Theory highlighted key mechanisms and contextual factors.

**Areas of Agreement:**

Ten interventions from 9 papers, including 371 young people, were included. Studies identified organizational demands, lack of implementation strategy and stigma as barriers to implementation, and clear training and plans for implementation as facilitators.

**Areas of Controversy:**

No standardized implementation outcomes were used across papers so meta-analysis was not possible.

**Growing Points:**

Barriers and facilitators have been clearly identified across different settings.

**Areas Timely for Developing Research:**

Longitudinal studies can identify methods and processes for enhancing long-term implementation and considers ways to monitor and evaluate uptake into routine practice.

## Introduction

Although many children and young people grow up psychologically well, recently published data suggest that 18.0% of children aged 7–16 years, and 22.0% of 17–24 year olds, have a probable mental disorder.^1^ Internalizing disorders, such as anxiety and depression, are the most common in this age range.^2^ Left untreated, internalizing mental health disorders are related to poor health, social and academic outcomes, as well as higher levels of self-harm and drug abuse.^3^

Several cognitive-behavioural therapy treatments (CBT) for childhood internalizing disorders have been systematically evaluated and received empirical support,^4^ but there remains overwhelming demand for psychological support. Shorter, less intensive psychological interventions and those that can be delivered by practitioners with less formal training have been proposed as treatment options to increase the capacity of mental health services and reduce the need for more intensive, longer term treatment in the future.^5^ There is evidence that brief and low-intensity interventions are as efficacious for children and young people with anxiety and depression as standard face-to-face interventions^6^ and preliminary evidence of their cost-effectiveness.^7^

### Brief and low-intensity interventions

Research suggests that clinically meaningful benefit from CBT can be observed in six-to-eight sessions, and that brief, and even single-session interventions, are effective in reducing depression and anxiety outcomes in children and adolescents.^8^ Shorter duration interventions can be more readily and widely deployed, increasing access to treatment and reductions in therapist contact time can be less costly to health service providers.^7^ These interventions can be utilized as part of a ‘stepped care approach’, using CBT with minimal therapist assistance as a first line of treatment and then ‘stepping up’ care to therapist-directed treatment to address individualized client needs for those requiring additional treatment. Stepped care interventions are therefore designed to maximize resources by providing lower intensity and less costly approaches as a first line treatment.^9^

Although an evidence-base is building which demonstrates the benefit of brief interventions in other conditions, such as substance use disorders (See Sarkar et al., (2020)^10^ for a review and Johnson et al., (2011) for lessons for implementation^11^), stepped care approaches are not consistently used across internalizing disorders, demonstrating an implementation gap.

### The implementation gap

There have been many research trials testing interventions to improve youth mental health and well-being and despite these interventions yielding medium to large positive effects for mental health, implementation is rarely considered, often due to the substantial time and additional funding it requires. Implementation strategies are defined as methods or techniques used to enhance the adoption, implementation and sustainability of a clinical programme or practice.^12^ The implementation gap is a term describing the time lag between research evidence and its integration into clinical practice or the gap between current best evidence and evidence-based practice.^13^ There remains an implementation gap for brief and low-intensity interventions in child and adolescent settings.

Implementation science seeks to understand how to best facilitate the use of evidence-based practices or interventions into routine practice and developing strategies to overcome barriers and increase the pace and effectiveness of implementation remains a high research priority.^14^ It is beneficial to consider barriers and facilitator to implementation with established theoretical frameworks. One such framework is Normalization Process Theory (NPT), which addresses the factors that are needed for successful implementation and the integration of interventions into routine practice.^15^ This can help offer explanation of how the intervention can become embedded into routine practice, i.e. how it is ‘normalized’. It is made up of four main components which overlap and interlink with each other and the wider context of the intervention. ‘Coherence’ explores whether the intervention makes sense and if it is easy to describe; ‘cognitive participation’ assesses whether target users will use the intervention; ‘collective action’ looks at how compatible the intervention is with existing work practices and ‘reflexive monitoring’ takes account of how participants reflect on or appraise the intervention.

### Aims

This review aimed to identify barriers and facilitators to implementing brief and low-intensity psychological interventions for children and young people with internalizing disorders or symptoms. Specifically, it aimed to identify studies that addressed methods, factors and/or processes which have enabled or prevented brief and/or low-intensity psychological interventions for children and young people to be successfully adopted and/or sustained.

## Methods

The review was registered on the International prospective register of systematic reviews (PROSPERO) in January 2022 (CRD42022307367) and Realist And MEta-narrative Evidence Syntheses: Evolving Standards (RAMESES) standards were adopted^16^ and provided in Appendix 1.

Rapid realist methodology was used to synthesize the research to better understand the mechanisms of the interventions in particular contexts and settings.^17^ This methodology is useful for time-sensitive and/or emerging issues, of interest to policy makers and applies a realist approach to knowledge synthesis.^18^

This review used the four components of NPT to guide data extraction and identify both helpful and obstructing factors, processes and methods for implementing brief and/or low-intensity psychological factors for children and young people with mental health difficulties.

### Search strategy

Searches were undertaken independently by two reviewers (A.R. and S.C.). PsycInfo, EMBASE and Medline were searched from inception to March 2022. The search terms were categorized into 3 primary areas: (i) Implementation, (ii) Mental health and (iii) Child and adolescent. The full search strategy is provided in Appendix 2. A manual search for grey literature on Google Scholar was also conducted and citation lists and reference lists of identified papers were searched for relevant papers.

### Study eligibility

Studies from clinical and non-clinical (e.g. schools) contexts from any country were included. Studies must have investigated the implementation of a brief and/or low-intensity psychological intervention.

For the purposes of this review, the definitions of brief and/or low-intensity interventions have been taken from a recent definition paper.^19^ Treatment for children and young people are defined as ‘brief’ if lasting for 50% of recommended therapy contact time as suggested in the National Institute for Clinical Excellence (NICE) guidelines.^19^ Low-intensity interventions require less therapeutic input that other treatment options can be provided by trained practitioners or supporters with less specialist training, usually have less than 6 hours of contact time and sessions typically last 30 minutes or less, often utilizing self-help or internet-based materials.^19^ Group interventions may last for 6–9 hours of therapist contact time, as per NICE guidelines.^20^^,^^21^

### Eligibility criteria

The psychological intervention investigated must have been (i) delivered to children and/or young people between 5 and 25 years, (ii) for children and/or young people with internalizing mental health difficulties and (iii) a brief and/or low-intensity treatment (as defined above). Included papers must have explored or identified methods, factors and/or processes for the adoption, implementation or sustainability of brief psychological interventions as one of the study outcomes. Studies of any design were included. There were no additional exclusion criteria.

### Data collection and analysis

Study selection was performed by A.R. S.C. screened a random 25% subset at title and abstract phase and full text screening phase to check for reliability.

### Data extraction

MS Excel was used to create a data extraction table. Extracted variables included title, date published, author, design, sample size, demographics, setting, intervention details (including total number of sessions and session length), primary and secondary outcomes, notes on coherence, cognitive participation, collective action, reflexive monitoring, study results, major limitations and conclusions.

### Quality assessment

Quality assessment of included articles was conducted using the Mixed Methods Appraisal Tool.^22^ This tool was appropriate to use as it allowed for simultaneous evaluation of different study designs. Two researchers (A.R. and S.C.) independently scored each article. The scoring was based on 5 criteria points where the researcher had to answer ‘yes’ or ‘no’ to each point. Articles scoring zero to one are reported as ‘low’ quality, two to four as ‘medium’ quality and five as ‘high’ quality.

### Narrative synthesis

Narrative synthesis^23^ was conducted independently by two reviewers (A.R. and S.C.). Researchers conducted preliminary analysis separately by extracting the descriptive characteristics of the studies and their reported barriers and facilitators. Researchers then came together and compared and organized findings to identify common themes across and within the studies.

## Results

**Fig. 1:**
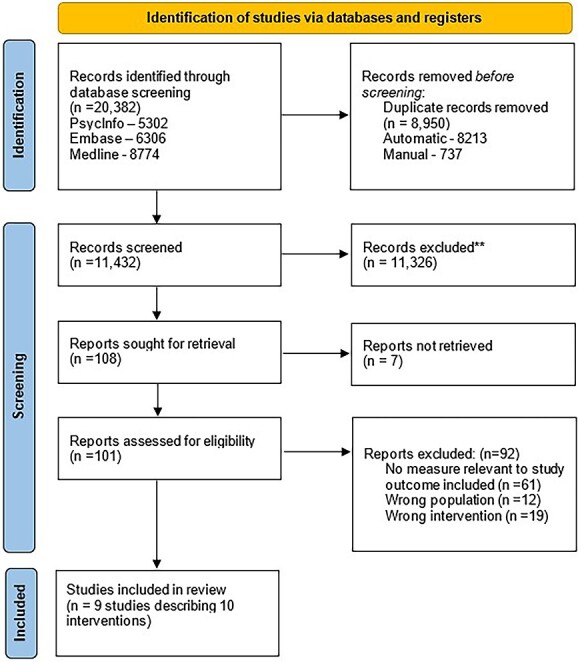
PRISMA diagram.

Out of 11 432 papers screened, 9 studies including 10 interventions were included in the analysis. This is displayed in Fig. 1. Studies investigated barriers and facilitators to implementing brief and/or low-intensity psychological interventions for 371 young people, delivered by 99 therapists.

### Study characteristics

Seven of the included interventions were delivered in the USA.^4^^,^^24–28^ The remaining studies were conducted in the UK,^29^ Australia^30^ and one paper explored the implementation of their intervention used across the world.^31^

Studies presented findings for a range of different low-intensity psychological interventions, including those for children and young people with anxiety,^4^^,^^24^^,^^25^^,^^28^ depression,^4^^,^^26–28^^,^^31^ eating problems^31^ and stress.^29^^,^^30^  Table 1 displays the intervention characteristics and study summaries are available in Appendix 3.

#### Study design and risk of bias

Three studies used qualitative methods,^26^^,^^27^^,^^29^ three used quantitative methods^25^^,^^28^^,^^30^ and one used mixed methods.^24^ The other two studies included a review of randomized control trials^31^ and a review book chapter which provided author reflections following the implementation of two low-intensity interventions.^4^

The mean quality rating was 2.67 (standard deviation 2.12), reported as ‘medium’. The quality of two of the qualitative studies was high,^26^^,^^29^ with rigorous data collection methods and coherent analysis and interpretation. The other qualitative study was low in quality due to unclear research questions, instead reporting outcomes as a single case study.^27^ The quantitative and mixed methods papers were of medium quality as it was not possible to deduct if the measures used were appropriate,^24^^,^^25^^,^^28^^,^^30^ and the final two papers were unable to be quality assessed due to their study design.^4^^,^^31^

#### Narrative synthesis

Papers identified different barriers and facilitators to implementation. These were narratively synthesized into 5 themes: organizational demands; the intervention and how it was explained; training and communication; implementation strategy and stigma. Some of these factors are fixed, and therefore difficult to change (e.g. financial demands of an organization) and others are adjustable (e.g. implementation strategy and intervention training). Stigma is considered as an external factor which is not implementation specific, however can be a barrier to intervention uptake.^32^ Themes are displayed in Table 2.

**Table 1:** Included studies with intervention details

**Author, year of publication, country**	**Intervention name**	**Intervention description**	**Young person demographics**	**Intervention components**	**Intervention deliverer**	**Brief or low intensity intervention?**	**Quality assessment score**
Becker, C. B. (2017),^31^ International	The Body Project	Small-group intervention for adolescent girls with negative affect and eating disorder behaviours.	Adolescent girls with negative affect and eating disorder behaviours	Single session 4-hour intervention delivered in a small group setting	Trained staff	Brief and low intensity	Not appliable (review paper)
Borschuk, A. P., et al. (2015),^27^ USA	Brief cognitive behavioural therapy (CBT)	7 sessions of CBT to reduce depressive symptoms	16-year-old female with depressive symptoms and suicidal behaviour	7 sessions lasting 30 minutes	Primary care physician	Brief	Low
Chu, B. C., et al. (2015),^26^ USA	Primary and Secondary Control Enhancement Training (PASCET)	The PASCET programme is an eight-session cognitive-behavioural intervention guided by a detailed therapist’s manual.	Children aged 8–12 years with depression	8 group sessions of around 50 minutes.	Two co-therapists with clinical training	Low intensity	High
Fox, J. K., et al. (2014),^4^ USA	Cool Kids	Cognitive-behavioural group intervention for children with anxiety	7–12 year old children with anxiety symptoms	8 group sessions lasting 1 hour	Trained therapist	Brief and low intensity	Not applicable (review chapter)
Fox, J. K., et al. (2014),^4^ USA	Skills for Academic and Social Success (SASS)	Cognitive-behavioural group treatment for delivery in school settings, for adolescents with social anxiety disorder	Adolescents with social anxiety disorder	12 group sessions lasting 40 minutes (Weekly group school sessions, two individual meetings, two parent meetings, two teacher meetings, four social events, and two booster sessions)	Psychologist and psychology graduate	Brief	Not applicable (review chapter)
Frank, H. E., et al. (2021),^24^ USA	Camp Cope A Lot	Computer-assisted cognitive behavioural therapy intervention for anxious youth	7–13 year olds with generalized anxiety disorder, social anxiety disorder and separation anxiety disorder.	12-session computer assisted CBT intervention f based on the Coping Cat programme	Providers did not need prior mental health training and participation was voluntary	Low intensity	Medium
Jagiello, T., et al. (2022),^30^ Australia	Study without stress (SWOS)	SWOS is grounded in CBT, and skills were taught primarily through didactic group work and homework exercises	Self-identified distressed year 11 students (15/16 years)	8 sessions of 30 minutes	Teachers trained to deliver the programme by the school counsellor	Brief and low intensity	Medium
Koschmann, E., et al. (2019),^28^ USA	School based CBT	Weekly student CBT skills groups led by a school professional and attended by a coach	8–18 year olds who scored highly on depression or anxiety self-reported questionnaire (PHQ9 and GAD7)	10group sessions lasting for a single class period (approximately 50mins)	School staff and a coach	Brief	Medium
LoCurto, J., et al. (2020),^25^ USA	Modular CBT (M-CBT)	Adapted CBT, made up of modules which can be delivered in any order	6–18 year olds who all met criteria for primary anxiety disorder	12 individual sessions of 30–40 minutes. Seven core modules: psychoeducation, exposure, rewards, cognitive restructuring, problem-solving, somatic and relaxation skills and relapse prevention	Clinician led	Brief	Medium
McKeague, L., et al. (2018),^29^ UK	DISCOVER – How to handle stress	Self-referral school-based group intervention designed for stressed students in sixth form	16–19 year olds who wished to receive psychological help for emotional difficulties	1-day workshop, for up to 15 students	One clinical psychologist and two assistant psychologists	Brief and low intensity	High

**Table 2:** Themes identified as barriers and facilitators to implementation

**Themes**	**Subthemes**	Becker, C. B., et al. (2017)	Borschuk, A. P., et al. (2015)	Chu, B. C., et al. (2015)	Fox, J. K., et al. (2014)	Frank, H. E., et al. (2021)	Jagiello, T., et al. (2022)	Koschmann, E., et al. (2019)	LoCurto, J., et al. (2020)	McKeague, L., et al. (2018)
1. Organizational demands	Time			**−**			**−**		**−**	**−**
	Cost	**−**	**−**			**−**				
	Space		**±**	**−**	**±**	**±**				**±**
	Capacity	**±**	**−**			**−**	**−**		**±**	**+**
2. Intervention and how it is explained		**±**	**±**	**±**		**−**			**−**	**±**
3. Training and communication		**±**	**−**	**+**	**−**	**+**			**±**	**±**
4. Implementation strategy		**+**				**+**		**+**		
5. Stigma			**−**			**−**				

+ = facilitator, − = barrier, **±** = discussed as both a barrier and a facilitator.

##### Theme 1: Organizational demands

Organizational demands were identified as being made up of four subfactors: time, cost, space and capacity. Four papers highlighted that there was not enough time to deliver the intervention^25^^,^^26^^,^^30^ or to even receive the training.^29^ Three papers outlined that cost was a huge barrier to delivering the intervention; highlighting financial constraints at their specific service^29^; the cost of staff to deliver interventions^27^^,^^31^ and how to move beyond research funding.^31^ Space and location were discussed as both a barrier and a facilitator, with some papers acknowledging the limited space available to deliver the interventions,^24^^,^^26^^,^^29^ others recognizing the benefit of delivering interventions in the same location as primary care^27^ and others suggesting treatment in schools can offer a convenient location which is already confidential, familiar and secure.^27^^,^^29^ The final organizational demand identified was capacity, with many papers identifying that clinician turnover, competing responsibilities and administration demands were barriers to implementation,^24^^,^^25^^,^^27^ whereas smaller caseloads, task shifting and interventions being provided by external agencies could help facilitate incorporation to routine practice.^25^^,^^29^^,^^31^

##### Theme 2: Intervention and how it is explained

The characteristics of the intervention and how it was explained was another factor identified as both helpful and an obstacle to implementation. The lack of flexibility to tailor materials to individuals and modules and materials that were not user friendly made implementation more difficult^25^^,^^26^^,^^31^ along with general physician ambivalence about the intervention utility and purpose, and lack of information on the intervention itself.^27^^,^^29^ Papers also expressed concerns around the confidentiality of outcomes from the intervention.^24^ Other studies explored ways to make the intervention more engaging to staff, such as using case study examples, outlining clear target symptoms and expectations for young people and using self-referral or opt-in models.^27^^,^^29^^,^^31^

##### Theme 3: Training and communication

The need for training and communication was discussed as both a barrier and a facilitator to implementation. The lack of skilled providers giving the training,^31^ and insufficient training creates barriers.^25^^,^^29^ On the other hand, combining didactic training with coaching,^26^ building good partnership between trainers and providers^31^ and positive feedback processes with lots of encouragement between the trainers and providers with supervision offered^25^ were found to facilitate the intervention. It was also beneficial when trainers were competent and knowledgeable.^24^

##### Theme 4: Implementation strategy

Having a clear implementation strategy was identified as a key facilitator.^28^ Several papers explored the benefits of providing tailored materials,^31^ readiness planning and pre-implementation activities.^24^ Studies that had spent time preparing for implementation generally reported better uptake and sustainment of their intervention.

##### Theme 5: Stigma

Although not an explicit implementation factor, stigma was identified as a barrier to implementation. Studies reported that the fear of stigma and embarrassment surrounding mental ill health was stopping young people from accessing and receiving psychological treatment,^24^^,^^27^ creating a further obstacle for the implementation of brief and low-intensity psychological interventions for children and young people with emotional and behavioural difficulties. No specific recommendations for overcoming stigma were reported in the included studies.

## Discussion

As the prevalence of mental distress among children and young people increases, together with demand for services, brief and low-intensity psychological interventions have been proposed as beneficial treatment options. These treatments exist, but most people do not receive them.^33^ The aim of this paper was to systematically review factors affecting the implementation of such interventions. The literature retrieved articles from different settings which, after using principles from the NPT framework^15^ during data extraction, identified common mechanisms that promoted or impeded implementation.

Key barriers included organizational demands such as financial concerns; staff turnover and time restraints; poor training and communication about the intervention; and stigma around mental health treatment. Most papers highlighted economic barriers which is in line with previous research, identifying how the costs of implementing evidence-based interventions can drive decision-making by service providers.^34^ Including information on financial implications, whether the cost-effectiveness of the intervention, or suggestions on how to implement efficiently using existing resources, can help with intervention adoption. Innovations that have a clear advantage in either effectiveness or cost-effectiveness are more easily adopted and implemented.^35^

Staff issues were also reported across multiple studies. In particular poor retention of individuals who had been specifically trained in intervention delivery, or those championing the intervention, created a considerable barrier to sustainability. This is consistent with research conducted in schools, indicating that these barriers may be central to sustainability irrespective of location.^36^ It may not be possible to overcome some of the central problems with staff turnover in mental health services, such as lack of career and job development and fixed-term contracts.^37^ However, there is suggestion that a ‘Train-the-Trainer’ model could be used to great benefit, ensuring staff on short-term job contracts are continually trained.^38^

Within the wider psychology literature, stigma is a known barrier to accessing mental health services.^32^ There is ongoing discussion over the best ways to engage young people in psychological interventions to avoid discrimination and embarrassment and potential suggestion that interventions could educate on strategies to alleviate the risk and/or impact of stigma.^39^

Key facilitators identified in this review were the availability of an implementation strategy, building good relationships between trainers and providers and careful consideration of intervention location. Papers highlighted the value in preparing for implementation. A multifaceted implementation strategy targeting multiple relevant determinants can be effective in initiating and sustaining implementation; however, methods used to select implementation strategies are not often well-described and no specific method or guideline has been proven superior. Allocating time for pre-implementation activities can facilitate implementation as it allows a trainee–trainer relationship to build which can enhance education and communication.^29^

Most papers discussed the location of the intervention. Locating services in primary care is considered hugely beneficial, and in line with the ongoing efforts to promote integrated care. Where interventions are delivered in schools, this can be a familiar setting which is convenient (less time away from routine for appointments) and a less ‘clinical’ environment, although both educational and clinical locations raise questions around room bookings and physical location restraints.^36^

### Strengths and limitations

This paper identified a broad range of brief and low-intensity interventions which are currently being delivered to children and young people with emotional difficulties. This allowed for a wide exploration of barriers and facilitators to their implementation, which were assessed using varied measures. Some papers gave a reflective account of problems faced and lessons learnt, some used mixed methodology and others had quantitative measures of uptake and sustainability. Despite the studies being heterogenous, similar barriers and facilitators were identified, suggesting that similar themes exist across locations, and conclusions can be applicable to other settings.

There were also some limitations. Most studies (7/10) were conducted in the USA, which uses a specific model of healthcare and insurance and thus barriers may reflect this. All studies where location was accurately reported were completed in high-income countries, often where research is recognized and possible to be implemented (albeit slowly) amongst healthcare and education settings. Although NPT components were used to guide the identification of themes, the results did not map onto the theory.

Furthermore, implementation was rarely the sole focus of the included studies. Although many studies from the initial search mentioned implementation in their title and abstract, there is discrepancy in how the term is used and gaps in reporting evidence of the sustainability of interventions. There also remains concerns over the psychometric quality of existing instruments.

### Recommendations

This review has identified clear areas of focus for implementation which can be translated across different therapeutic settings. Organizational factors are often fixed and difficult to change, however, identifying barriers such as cost can help inform researchers at intervention development stage, ensuring that implementation is considered from the start.^40^ Engaging wider teams and networks rather than reliance on individual members of staff to drive forward sustainment can help overcome staff turnover issues. Building relationships with service deliverers to understand work capacities and responsibilities can initiate understanding of how interventions can best fit in practice. Utilizing an implementation strategy can help guide interventions from research ideas into clinical practice and planning for such strategy should be considered as early as possible.

Future research should seek to report implementation outcomes and studies should be conducted longitudinally to assess the effect and impact of individual barriers and facilitators over time. It would also be beneficial to compare longitudinal implementation studies of all psychological interventions with brief and low-intensity treatments to assess if any specific adoption processes are valuable.

## Conclusion

This review identified specific factors, methods and processes which have enabled brief and low-intensity psychological interventions for children and young people with internalizing disorders to be successfully adopted and/or sustained. These should be considered from the start of intervention inception, up to its implementation and beyond.

## Authors’ contributions

Anna Roach (Conceptualization, Data curation, Formal analysis, Investigation, Methodology, Project administration, Writing—original draft, Writing—review & editing), Sophie Cullinan (Data curation, Formal analysis, Writing—review & editing), Roz Shafran (Conceptualization, Methodology, Supervision, Validation, Writing—review & editing), Isobel Heyman (Supervision, Writing—review & editing), and Sophie Bennett (Conceptualization, Formal analysis, Investigation, Supervision, Writing—review & editing).

## Data availability

The authors confirm that the data supporting the findings of this study are available within the article and its supplementary materials.

## Supplementary Material

Appendix_1_RAMESES_publication_standards_ldad001

Appendix_2_Final_search_strategy_ldad001

Appendix_3_Study_characteristics_of_included_studies_ldad001
