# Supplementary material for: Implementing brief and low-intensity psychological interventions for children and young people with internalizing disorders: a rapid realist review
Source: Br Med Bull. 2023 Jan 28;145(1):120–31. doi: 10.1093/bmb/ldad001 (PMC10075242; doi:10.1093/bmb/ldad001)
Supplement: Appendix_2_Final_search_strategy_ldad001 [file appendix_2_final_search_strategy_ldad001.docx]

**Appendix 2: Final search strategy**

| **PsycInfo (via OVID)** | (implement* or adopt* or sustain* or promot* or inhibit* or feasibility or suitab* or acceptability or application or integration or routine practice or program* evaluation or translat* or innovat* or guideline* or guidance or uptake or utilis* or utiliz*).ti,ab.  (((brief or low intensity or short or minimal) adj (psychological intervention* or psychosocial intervention* or psycho-social intervention* or psychological treatment* or psychosocial treatment* or psycho-social treatment* or therap* or psychotherap*)) or psycho-education or psychoeducation or CBT or cognitive behavio* therap* or self-help or (self management and (psychological or psychosocial or psycho-social or therap*)) or cognitive therap* or behavio?ral therap*).ti,ab.  (Child* or Young people or Adolesc* or Youth or Teen* or Minor* or Juvenile* or Young person or Student* or High school or Middle school or Secondary school or Young women or young woman or Young men or Young males or Young females or Boy or boys or Girl* or Pediatric or paediatric or Young adult or Pupil*).ti,ab. |
| --- | --- |
| **EMBASE (via OVID)** | (implement* or adopt* or sustain* or promot* or inhibit* or feasibility or suitab* or acceptability or application or integration or routine practice or program* evaluation or translat* or innovat* or guideline* or guidance or uptake or utilis* or utiliz*).ti,ab,kf.  (((brief or low intensity or short or minimal) adj (psychological intervention* or psychosocial intervention* or psycho-social intervention* or psychological treatment* or psychosocial treatment* or psycho-social treatment* or therap* or psychotherap*)) or psycho-education or psychoeducation or CBT or cognitive behavio* therap* or self-help or (self management and (psychological or psychosocial or psycho-social or therap*)) or cognitive therap* or behavio?ral therap*).ti,ab,kf.  (Child* or Young people or Adolesc* or Youth or Teen* or Minor* or Juvenile* or Young person or Student* or High school or Middle school or Secondary school or Young women or young woman or Young men or Young males or Young females or Boy or boys or Girl* or Pediatric or paediatric or Young adult or Pupil*).ti,ab,kf. |
| **Medline (via OVID)** | (implement* or adopt* or sustain* or promot* or inhibit* or feasibility or suitab* or acceptability or application or integration or routine practice or program* evaluation or translat* or innovat* or guideline* or guidance or uptake or utilis* or utiliz*).ti,ab,kf.  MESH:  Implementation Science/ or Health Plan Implementation/  Feasibility Studies/  Program Evaluation/  "diffusion of innovation"/  Practice Guidelines as Topic/  (((brief or low intensity or short or minimal) adj (psychological intervention* or psychosocial intervention* or psycho-social intervention* or psychological treatment* or psychosocial treatment* or psycho-social treatment* or therap* or psychotherap*)) or psycho-education or psychoeducation or CBT or cognitive behavio* therap* or self-help or (self management and (psychological or psychosocial or psycho-social or therap*)) or cognitive therap* or behavio?ral therap*).ti,ab,kf.  MESH:  Psychotherapy, Brief/  Psychosocial Intervention/  Self Care/  Cognitive Behavioral Therapy/  (Child* or Young people or Adolesc* or Youth or Teen* or Minor* or Juvenile* or Young person or Student* or High school or Middle school or Secondary school or Young women or young woman or Young men or Young males or Young females or Boy or boys or Girl* or Pediatric or paediatric or Young adult or Pupil*).ti,ab,kf.  MESH:  adolescent/ or child/ |
