# Supplementary material for: Implementing brief and low-intensity psychological interventions for children and young people with internalizing disorders: a rapid realist review
Source: Br Med Bull. 2023 Jan 28;145(1):120–31. doi: 10.1093/bmb/ldad001 (PMC10075242; doi:10.1093/bmb/ldad001)
Supplement: Appendix_3_Study_characteristics_of_included_studies_ldad001 [file appendix_3_study_characteristics_of_included_studies_ldad001.docx]

| **Author, year of publication, country** | **Study design** | **Sample size** | **Clinical or non-clinical setting** | **Setting specifics** |
| --- | --- | --- | --- | --- |
| Becker, C. B. (2017)^36^, International | Review paper of lessons learnt from other trials and implementation studies | Not specifically reported, suggested the intervention has been delivered to over 3.5 million adolescent girls | Non-clinical | Universities |
| Borschuk, A. P., et al. (2015)^32^, USA | Case study | N=1 | Clinical | Paediatric Primary Care  Setting |
| Chu, B. C., et al. (2015)^31^, USA | Qualitative interview study | N=23 | Clinical | Outpatient public  mental health clinics |
| Fox, J. K., et al. (2014)^7^, USA | Review chapter | N=162 (across three research studies) | Non-clinical | Schools |
| Frank, H. E., et al. (2021)^29^, USA | Observational study (part of larger trial) | 38 schools | Non-clinical | School |
| Jagiello, T., et al. (2022)^35^, Australia | Implementation trial | n=80 students and n=6 programme facilitators | Non-clinical | School |
| Koschmann, E., et al. (2019)^33^, USA | Observational cohort study | N=105 students and n=17 school professionals | Non-clinical | School |
| LoCurto, J., et al. (2020)^30^, USA | Questionnaire study | N=43 school based clinicians | Non-clinical | School |
| McKeague, L., et al. (2018)^34^, UK | Qualitative study | N=21 students and n=10 school staff | Non-clinical | School |

**Appendix 3: Study characteristics**
